# Social desirability and self-reported health risk behaviors in web-based research: three longitudinal studies

**DOI:** 10.1186/1471-2458-10-720

**Published:** 2010-11-23

**Authors:** Rik Crutzen, Anja S Göritz

**Affiliations:** 1 CAPHRI, Maastricht University, Maastricht, The Netherlands; 2 Work, Industrial & Organizational Psychology, University of Würzburg, Würzburg, Germany

## Abstract

**Background:**

These studies sought to investigate the relation between social desirability and self-reported health risk behaviors (e.g., alcohol use, drug use, smoking) in web-based research.

**Methods:**

Three longitudinal studies (Study 1: *N *= 5612, 51% women; Study 2: *N *= 619, 60%; Study 3: *N *= 846, 59%) among randomly selected members of two online panels (Dutch; German) using several social desirability measures (Marlowe-Crowne Scale; Balanced Inventory of Desirable Responding; The Social Desirability Scale-17) were conducted.

**Results:**

Social desirability was not associated with self-reported current behavior or behavior frequency. Socio-demographics (age; sex; education) did not moderate the effect of social desirability on self-reported measures regarding health risk behaviors.

**Conclusions:**

The studies at hand provided no convincing evidence to throw doubt on the usefulness of the Internet as a medium to collect self-reports on health risk behaviors.

## Background

This work sought to investigate the relation between social desirability and self-reported health risk behaviors (e.g., alcohol use, drug use, and smoking) in web-based research. Self-report measures are a common way of gathering data in research on health risk behaviors. In several commonly used planning models of health promotion [1,2], self-reports are used in several phases, for example, in the problem analysis (e.g., behavioral diagnosis) and in the evaluation of interventions (e.g., effectiveness). In tailored interventions, self-reports are used to tailor the intervention to respondents' behavior and determinants of this behavior [3,4]. One reason of why self-reports are used in research on health risk behaviors is that they require fewer resources (e.g., financial, logistical) and have higher specificity (e.g., quantity/frequency measures) compared to bio-medical measures such as hair testing and urine screening for drug use or an air carbon monoxide monitor for smoking. Another reason of why self-reports are used in research is that interventions are nowadays increasingly delivered through the Internet [5,6]. Internet-delivered interventions often rely on self-reports, because bio-medical measures are not consonant with grounds to deliver interventions through the Internet such as accessibility (24/7 worldwide), convenience (e.g., participating in the comfort of one's own home), and anonymity (e.g., no human contact).

A study by Kreuter, Presser, and Tourangeau [7] indicated an increase in the reporting of sensitive information in web-based questionnaires relative to conventional telephone interviewing, whereas another study found no differences when comparing web-based with paper-and-pencil questionnaires [8]. On the one hand, some researchers stated that the social distance [9] and the impersonal nature of the Internet might inhibit trust development [10]. Link and Mokdad [11], for example, found the use of web-based research with the general public to be problematic (e.g., because of obtaining considerable variation in the estimates for heavy drinking). On the other hand, previous research indicated that smoking behavior can indeed be reliably assessed by self-reports obtained via the web [12,13]. Furthermore, McCabe and colleagues [14] provide strong evidence that web-based research can be used as an effective mode for collecting alcohol and other drug use data.

While some studies speak in favor of assessing alcohol use and addiction severity via the web [15,16], others found underreporting of undesirable behaviors, such as drug use and alcohol use [17]. Social desirability may provide an explanation for these different findings. Social desirability is the tendency of respondents to distort self-reports in a favorable direction, for example, by providing responses that - to their belief - are consistent with social norms and expectations [18].

There has been a long discussion in the literature whether social desirability is a personality trait or a situational strategy [19]. Previous research using latent state-trait models indicates that the largest proportion of variance in responses is attributable to differences in the trait. A small but significant proportion of variance is due to situation-specific conditions [20]. A condition that tends to enhance the possibility of social desirability bias is a highly sensitive topic [21]. Moreover, significant relationships between social desirability and self-reports of risk-taking behavior have been revealed previously [22]. Hence, it is reasonable to assume that many areas of public health, particularly self-reports of health risk behaviors, are prone to social desirability bias. If self-reported measures are indeed influenced by social desirability, controlling for social desirability may remove some of the error due to the use of self-report measures and therewith improve the validity of these measures [23].

Previous research found minimal evidence of an influence of social desirability on scores from two self-report measures of measuring physical activity in young adults [24] and no evidence for a social desirability bias with a self-report condom use scale [25]. Nevertheless, these studies were not web-based, thereby ignoring the social distance and impersonal nature of the Internet. Mode comparison studies (i.e., in which web-based assessment is compared with, for example, paper-and-pencil assessment [26,27] or with telephone interviewing [28]) generally have relied on one of three different designs: randomization after recruitment (true experimental design), randomization before recruitment (where there may be differences in response between modes), and a test-retest design (where respondents need to answer questions in two or more modes consecutively). A recent report on online panels by the American Association for Public Opinion Research [29] concluded that, regardless of design, there were higher reports of socially undesirable attitudes and behaviors in self-reported web-based questionnaires than in face-to-face interviews. For example, web-based questionnaires yielded higher reports of smoking [30] and alcohol use [31]. These studies compared different modes regarding self-reports of health risk behaviors (e.g., differences in prevalence rates) and attributed the studies' results to characteristics of that mode. In other words, these studies assumed that certain modes lead to more or less socially desirable responding. Hence, the focus of these studies was not on the influence of social desirability itself. It is possible, for example, that there were other factors, besides social desirability, that led to differences in reports of health risk behaviors. In contrast to the work at hand, these studies did not investigate whether differences in social desirability resulted in differences in self-reports of health risk behaviors.

If social desirability is found out to be an issue in web-based research, this would raise concerns about the validity of web-based research on health risk behaviors. Therefore, in the work at hand we were specifically interested in the relationship between social desirability and the self-reporting of health risk behaviors in web-based research. We investigated the association between social desirability measures and self-reported health risk behaviors. Hence, the following research question was put forward:

To what extent is social desirability associated with self-reported health risk behaviors in web-based research?

Because of the social distance [9] and the impersonal nature of the Internet [10], we did not expect social desirability to have a biasing influence in web-based research on health risk behaviors. Additionally, we investigated potential moderating effects of socio-demographics on the effects of social desirability on self-reports of health risk behaviors. In line with a meta-analysis about social desirability distortion [32], we did not expect any moderating effects of socio-demographics.

Due to the explorative nature of our research, we collected data in three longitudinal studies among randomly selected members of two online panels using several social desirability measures. In the first study, the traditional social desirability measure was used: the Marlowe-Crowne Scale [33]. For this measure, items were selected from personality questionnaires that described behaviors that were highly desirable but unlikely to be true or undesirable but likely to be true. High scorers on the Marlowe-Crowne Scale are more amendable to social influence compared to low scorers. Therefore, higher scores are probably related to impression management; a tendency to intentionally distort one's self-image to be perceived favorably by others [34].

Gawronski and colleagues [35] argued, however, that the Marlowe-Crowne Scale may be too general to capture motivational distortions in self-reports and a more differentiated social desirability measure distinguishing between self-deception and impression management may be needed. Self-deception is an unintentional propensity to portray oneself in a favorable light, manifested in positive but honestly believed self-descriptions [34]. Impression management, by contrast, is people's tendency to intentionally distort their self-presentation to be perceived favorably by others The Balanced Inventory of Desirable Responding (BIDR) [36] appeared to be useful for our purposes, since this measure has two subscales measuring both self-deception (BIDR-SE) and impression management (BIDR-IM). The BIDR-IM was used in the second study because this subscale is more closely related to the Marlowe-Crowne Scale and is deemed to be instrumental for our purposes.

Another critique on the Marlowe-Crowne Scale says this scale reflects the social standards of the late 1950 s (e.g., "I am always courteous, even to people who are disagreeable.") and is less appropriate to be used nowadays [37]. To remedy this limitation, the Social-Desirability Scale-17 (SDS-17) was developed [37]. This is a new scale in the Marlowe-Crowne style, but with up-to-date contents. To avoid falling prey to potential problems of validity with the Marlowe-Crowne Scale, in the third study, we used the SDS-17 next to both subscales of the BIDR. We hypothesized - in line with Stöber [37] - that the SDS-17 is more highly correlated with the BIDR-IM than with the BIDR-SE. Besides differences in correlations among scales, we did not hypothesize differences among the scales regarding their relationship to self-reports of health risk behaviors, since we did not expect social desirability to have an influence in web-based research on health risk behaviors in the first place.

## Study 1: Methods

A longitudinal study was conducted to investigate the relation between social desirability and self-reported health risk behaviors in web-based research. Data were collected through the LISS panel http://www.lissdata.nl. The reference population for the LISS panel is the Dutch speaking population permanently residing in the Netherlands. In co-operation with Statistics Netherlands addresses were drawn from the nationwide address frame. The sample from the population registers includes individuals who do not have Internet access. These participants were provided equipment to access the Internet via a broadband connection. Sample members with small band Internet access were provided with broadband [38]. There was no ethics approval for this study specifically, but for the umbrella project which was conducted by an external party (CentERdata; http://www.centerdata.nl/en). Relevant ethical safeguards were met with regard to the participant confidentiality and consent.

### Procedure and Respondents

Data on social desirability were collected between May 2008 and August 2008 (T1). In total, 8,722 panel members were invited. Of those, 6,808 initiated the questionnaire (response rate 78.1%) and 6,766 completed the social desirability measure (completion rate 99.4%). This initial sample of 6,766 panel members was re-invited - between November 2008 and December 2008 (T2) - to complete the follow-up measures on health risk behaviors. Of those, 5,635 initiated the questionnaire (response rate 83.3%) and 5,612 completed the health risk behavior measures (99.6%). This resulted in a final sample of 5,612 respondents, who were included in the analyses (Table 1).

**Table 1:** Sample characteristics (Study 1; *N = *5,612)

Socio-demographics		
Age		M = 46.9 (16.0)
Sex	Female	51.2%
	Male	48.8%
Personal net monthly income	(in Euros)	Median = 1,300
Level of education	Primary school	9.7%
	Intermediate secondary education	26.4%
	Higher secondary education/preparatory university education	11.0%
	Intermediate vocational education	23.1%
	Higher vocational education	22.3%
	University	7.5%

Social desirability (Marlowe-Crowne)		M = 5.9 (1.5) (Scale: 0-10)

Health risk behaviors		
	
Current behavior	Alcohol use	72.7%
	Sedatives	4%
	Soft drugs	3%
	XTC	0.5%
	Hallucinogens	0.2%
	Hard drugs	0.5%
	Smoking	35.6%
	
Frequency	Alcohol use	M = 3.4 (2.2)
	Sedatives	M = 13.3 (27.6)
	Soft drugs	M = 13.4 (26.0)
	XTC	M = 1.5 (0.9)
	Hallucinogens	M = 1.2 (0.6)
	Hard drugs	M = 2.9 (3.3)
	Smoking	M = 11.6 (8.1)

### Measures

#### Socio-demographics

Besides age and sex, two predictors of socio-economic status were measured: personal net monthly income (in Euros) and level of education. A detailed description of the procedure that we used to determine personal net monthly income can be found elsewhere [39]. Level of education was categorized according to the definitions of Statistics Netherlands, resulting in six categories: primary school, intermediate secondary education (US: junior high school), higher secondary education/preparatory university education (US: senior high school), intermediate vocational education (US: junior college), higher vocational education (US: college), and university. Socio-demographics of all panel members were known in advance. This provided the opportunity to conduct attrition analyses regarding socio-demographic variables.

#### Social desirability

Social desirability was measured by the shortened version of the Marlowe-Crowne Scale [33], which has been validated previously [40]. This scale consists of ten true/false statements, e.g., "I am always courteous, even to people who are disagreeable". The scale score ranges from zero to ten. A high score indicates a high tendency to provide socially desirable responses.

#### Health risk behaviors

Two aspects with regard to health risk behaviors were assessed: (1) current behavior and (2) behavior frequency among those who carried out the behavior in question. Current behavior was assessed for alcohol use (Have you had a drink containing alcohol during the last seven days), drug use (Have you used ... over the past month?), and smoking (Do you smoke?). Sedatives (e.g., valium), soft drugs (e.g., hashish, marijuana), XTC, hallucinogens (e.g., LSD, magic mushrooms), and hard drugs (e.g., cocaine, heroine) were included as separate items regarding drug use. XTC was considered as a separate category because of its high rate of use in the Netherlands [41]. Behavior frequency was also assessed for alcohol use (On how many of the past seven days did you have a drink containing alcohol?), drug use (How often have you used ... over the past month?), and smoking (How many cigarettes (including rolling tobacco) do you smoke on average per day?). According to the obtained self-reports of current behavior, sedatives, soft drugs, XTC, hallucinogens, and hard drugs were included as separate items regarding frequency of drug use.

### Analyses

First, attrition analyses, by means of t-tests and χ^2^-tests, were conducted to test for possible differences between retainees and drop-outs with regard to socio-demographics. Second, multiple regression analyses were conducted. Current behavior (dichotomous variables; logistic regression analyses) and its frequency (linear variables; linear regression analyses) were the dependent variables. The linear dependent variables were subjected to Box-Cox-transformations to meet the assumption of normality [42]. Age, sex, personal net monthly income, education, and social desirability (at T1) were included in the model as predictors of the dependent variables (at T2). Moreover, interaction terms between socio-demographics (i.e., age, sex, personal net monthly income, and education) and social desirability were added to test for possible moderating effects [43]. Odds ratios were converted into Cohen's *d *(as described by Chinn [44]) to be able to report standardized effect sizes.

## Study 1: Results

Retainees in the final sample did not differ in sex (χ^2^(1, *N *= 6,603) = .23, *p *= .64), personal net monthly income (t(6,285) = .72, *p *= .47), and education (χ^2^(5, *N *= 6,603) = 10.24, *p *= .07) from panel members who dropped-out. Those who dropped-out, however, were younger than those who completed both questionnaires (42.1 versus 46.9 years, t(6,601) = 9.16, *p *< .01).

Social desirability was not associated with reported current behavior or behavior frequency (Additional file 1). The only exception was a positive effect of social desirability on the self-reported use of hard drugs (OR = 4.86, *p *< .01, 95% CI = 1.88-12.56). The broad confidence interval reflects the small number of participants concerned [45], since only 0.5% of our sample reported having used hard drugs over the past month (Table 1).

Most interactions terms between socio-demographics and social desirability were not significantly associated with current health risk behaviors or health risk behavior frequencies. The only exception was an interaction between education and social desirability: Those at the lowest educational level (i.e., primary school) and a high social desirability score were more likely to report having used hard drugs over the past month (OR = 2.47, *p *< .05, 95% CI = 1.02 - 5.99). The broad confidence interval reflects the small number of participants concerned regarding hard drug use.

## Study 2: Methods

A second study was conducted to investigate the robustness of the first study's findings on another large sample, with another social desirability measure and implementing a larger time lag between the measurement of social desirability and self-reported health risk behaviors.

The Balanced Inventory of Desirable Responding (BIDR) [36], which has been validated in Germany [46], was used to measure social desirability. Furthermore, a different online panel was used than in the previous study, namely the WiSo-Panel http://www.wisopanel.uni-erlangen.de. This panel holds demographically heterogeneous participants from all walks of life, of which 99% are German speaking Germans, Austrians, and Swiss. People have been recruited for this panel from different sources using a wide range of methods - both probabilistic [47] and non-probabilistic (e.g., newsletters, participants in one-shot web-studies, word-of-mouth, search engines). This study was approved by the German Research Foundation, which included an approval of ethical aspects.

### Procedure and Respondents

Data regarding social desirability were collected in October and November 2008 (T1). In total, 5,857 panel members were invited by e-mail. Of those, 1,694 initiated the questionnaire (response rate 28.9%) and 1,438 completed the social desirability measure (completion rate 84.9%). The sample of who had completed the social desirability measure was re-invited - in December 2009 (T2) - to complete the follow-up measures regarding health risk behaviors. In between T1 and T2, 57 people had left the panel; therefore the remaining 1,381 panel members were invited to T2. Of those, 644 called up the questionnaire (response rate 46.6%), and of those who respondended, 619 completed the health risk behavior measures (completion rate 96.1%). This resulted in a final sample of 619 respondents (Table 2).

**Table 2:** Sample characteristics (Study 2; *N *= 619)

Socio-demographics		
Age		M = 39.1 (11.7)
Sex	Female	60.4%
	Male	39.6%
Level of education	No degree	1.3%
	Nine years of school	10.3%
	Vocational qualification	33.1%
	University qualification	36.2%
	University	18.4%
	Doctorate	0.6%

Social desirability (BIDR-IM)		M = 3.6 (1.0) (Scale: 1-7)

Health risk behaviors		
	
Current behavior	Alcohol use	59.0%
	Smoking	33.9%
	
Frequency	Alcohol use	M = 2.6 (1.8)
	Smoking	M = 16.3 (9.7)

### Measures

#### Socio-demographics

Age, sex, and level of education. Education was categorized in line with the German school system: no degree (i.e., only primary school), nine years of school (US: junior high school), vocational qualification (US: senior high school), university qualification (US: senior high school), university (US: Bachelor's and Master's degree), and doctorate (US: PhD). Socio-demographics of all panel members were known in advance. This provided the opportunity to conduct attrition analyses regarding socio-demographics.

#### Social desirability

Social desirability was measured by the impression management scale of the BIDR (BIDR-IM). Respondents are required to indicate their agreement with ten statements about themselves on a 7-point scale, with 1 denoting "fully disagree" and 7 denoting "fully agree". After reversing negatively keyed items, the score on this scale ranges from one to seven. A high score indicates a high tendency of impression management.

#### Health risk behaviors

Two aspects of health risk behaviors were assessed: (1) current behavior and (2) behavior frequency among those who carried out the behavior in question. Current behavior was assessed for alcohol use (Have you had a drink containing alcohol during the last seven days) and smoking (Do you smoke?). Behavior frequency was also assessed for alcohol use (On how many of the past seven days did you have a drink containing alcohol?) and smoking (How many ... do you smoke on average per day?). With regard to smoking, we added cigarettes and hand-rolled cigarettes to determine the number of cigarettes (including rolling tobacco) [48].

### Analyses

Attrition analyses and multiple regression analyses were comparable to those conducted in the first study.

## Study 2: Results

Retainees in the final sample did not differ in sex (χ^2^(1, *N *= 1,505) = 1.96, *p *= .16) from panel members who had dropped-out. Those who dropped-out, however, were younger than those who completed both questionnaires (35.0 versus 39.1 years, t(1,501) = 6.50, *p *< .001). Moreover, drop-outs were more likely to have a university qualification (46.1% versus 36.2%, OR = 1.51, *p *< .01, 95% CI 1.23 - 1.85).

Social desirability, as measured by BIDR-IM, was not associated with reported current behavior or behavior frequency (Table 3). Socio-demographics (i.e., age, sex, and education) did not moderate the effect of social desirability on self-reported health risk behaviors and their frequency.

**Table 3:** Effect of social desirability on self-reported health risk behaviors (Study 2; *N *= 619)

	**Current behavior**^**1**^		**Frequency**^**1**^	
	**Alc**	**Smo**	**Alc**	**Smo**

**Predictors**	***d***	***d***	**ß**	**ß**

Age	.00	.01	.28*	.26*
Sex	-.35*	.15	-.20*	.08
Education^2^	6.89	21.00*	.09	-.12

SocDes^3^	.00	-.43	-.10	-.50

Age × SocDes	.01	.00	-.03	.07
Sex × SocDes	.11	.10	.08	.15
Education × SocDes^2^	2.83	.06	-.05	.36

R^2^	.10	.12	.15	.11

^1^Alc = Alcohol use; Smo = Smoking; ^2^Wald statistic instead of Cohen's *d*; ^3^Social desirability; * *p *< .05

## Study 3: Methods

To throw more light on the issue of the results depending on the choice of scale, this study employed several social desirability measures.

A five-wave longitudinal study was conducted; the first four waves were used to measure social desirability and the fifth wave was used to obtain self-reports on health risk behaviors. Four separate waves were used to avoid contamination between different social desirability measures as well as to determine the re-test reliability of measuring social desirability. A random sample of the same panel but consisting of different panel members as in Study 2 was used. This study was approved by the German Research Foundation, which included an approval of ethical aspects.

### Procedure and Respondents

Data on social desirability were collected in November 2008 (T1), December 2008 (T2), March 2009 (T3), and April 2009 (T4). In total, 3,201 panel members were invited by e-mail. Of those, 2,037 initiated the questionnaire (response rate 63.6%), and of those responding, 1,829 completed the social desirability measure in T1 (completion rate 89.8%). In T2 we invited the remaining 3,168 panel members from the original sample. Of those, 1,875 initiated the questionnaire (response rate 59.2%), and of those responding, 1,733 completed the social desirability measure (completion rate 92.4%). In T3 we invited the then remaining 3,136 panel members from the original sample. Of those, 1,769 initiated the questionnaire (response rate 56.4%), and of those responding, 1,362 completed the social desirability measure (completion rate 77.0%). In T4 the then remaining 3,124 panel members from the original sample were invited. Of those, 1,630 called up the questionnaire (response rate 52.2%), and of those responding, 1,481 completed the social desirability measure (completion rate 90.9%). A total of 2,493 panel members completed at least one of the social desirability measures. Of this group, those 2,390 panel members who were still members of the panel were invited in December 2009 (T5) to complete self-reports on their health risk behaviors. Of those invited, 996 initiated the questionnaire (response rate 41.7%), and of those responding, 846 completed both health risk behavior measures (completion rate 84.9%). This resulted in a final sample of 846 respondents (Table 4).

**Table 4:** Sample characteristics (Study 3; *N *= 846)

Socio-demographics		
Age		M = 43.9 (11.7)
Sex	Female	58.7%
	Male	41.3%
Level of education	No degree	0.6%
	Nine years of school	11.3%
	Vocational qualification	34.4%
	University qualification	23.5%
	University	28.8%
	Doctorate	1.5%

Social desirability	BIDR-IM1	M = 3.9 (1.0) (Scale: 1-7)
	SDS-17	M = 10.2 (3.1) (Scale: 0-16)
	BIDR-IM2	M = 3.9 (1.1) (Scale: 1-7)
	BIDR-SE	M = 4.2 (0.8) (Scale: 1-7)

Health risk behaviors		
	
Current behavior	Alcohol use	66.1%
	Smoking	33.8%
	
Frequency	Alcohol use	M = 2.7 (1.9)
	Smoking	M = 15.4 (9.0)

### Measures

Socio-demographics and health risk behaviors were measured in the same fashion as in Study 2. The following social desirability measures were used:

#### *T1 *(BIDR-IM1)

Impression management scale of the BIDR (the same one used as in Study 2).

#### *T2 *(SDS-17)

Social-Desirability Scale-17, which has also been validated in Germany [37]. As recommended by Stöber [37], one item was deleted from the final version of the SDS-17, leaving sixteen true/false statements (e.g., "I never hesitate helping someone in case of emergency"). The scale score ranges from zero to sixteen, with a high score indicating a high tendency to give socially desirable responses.

#### *T3 *(BIDR-IM2)

Impression management scale of the BIDR (the same one used as in T1 to assess temporal stability of this scale between T3 and T1).

#### *T4 *(BIDR-SE)

Self-deceptive enhancement scale of the BIDR. Similar to the impression management scale (which is the other scale of the BIDR), respondents need to indicate their agreement with ten statements about themselves on a 7-point scale, with 1 denoting "fully disagree" and 7 denoting "fully agree". After reversing negatively keyed items, the score on this scale ranges from one to seven, with a high score indicating a high tendency of self-deceptive enhancement.

### Analyses

Attrition analyses and multiple regression analyses proceeded comparably to those conducted in the previous studies. Before conducting these analyses, however, Pearson correlation coefficients among the three social desirability measures were calculated (Table 5). These correlations were comparable in size to those in Musch and colleagues [46]. Moreover, the intercorrelations among the different social desirability measures followed an expectable pattern: Using the same measure at two time points (i.e., re-test reliability of BIDR-IM) yields the highest correlation (.74), followed by intermediate correlations between two different social desirability measures (.55 for SDS-17 and BIDR-IM1, .60 for SDS-17 and BIDR-IM2, .40 for SDS-17 and BIDR-SE), followed by the lowest correlations between two complementary scales that are supposed to capture different facets of social desirability (.28 for BIDR-IM1 and BIDR-SE, .40 for BIDR-IM2 and BIDR-SE). Moreover, the re-test reliability of BIDR-IM (. 74) was about as large as the internal consistency of the measurement at either time point (.74 for BIDR-IM1 and .72 for BIDR-IM2), which speaks to the quality of the measurement. To prevent multicollinearity from distorting results, separate regression models were created for each social desirability measure, resulting in four final models per dependent variable.

**Table 5:** Correlations between social desirability measures (Study 3; *N *= 846)

Wave	Measure	BIDR-IM1	SDS-17	BIDR-IM2	BIDR-SE
T1	BIDR-IM1 (α = .74)	-- N = 1820	.55* N = 1306	.74* N = 1048	.28* N = 1093
T2	SDS-17 (α = .72)		-- N = 1733	.60* N = 1057	.40* N = 1117
T3	BIDR-IM2 (α = .72)			-- N = 1358	.31* N = 1000
T4	BIDR-SE (α = .68)				-- N = 1477

* *p *< .01 (2-tailed)

## Study 3: Results

Panel members who dropped out were more likely to be women (63.2% versus 58.7%, χ^2^(1, *N *= 1,964) = 3.94, *p *< .05) and younger (42.2 versus 43.9 years, t(1,960) = 2.56, *p *= .01) than retainees in the final sample. Moreover, drop-outs were more likely to have a vocational qualification (34.4% versus 25.9%, OR = .48, *p *= .04, 95% CI .24 - .97).

By and large, the social desirability measures were not associated with self-reported current behavior or behavior frequency (Additional file 2). Moreover, the interactions terms between socio-demographics (i.e., age, sex, and education) and social desirability were not significantly associated with health risk behaviors or health risk behavior frequencies. The only exceptions were two interactions between education and the self-deceptive enhancement scale: Those at the higher educational level and a high unintentional propensity to portray oneself in a favorable light reported lower behavior frequency regarding alcohol use and smoking.

## Discussion

Three longitudinal studies revealed no meaningful associations between social desirability and self-reported health risk behaviors in web-based research. This is in line with our hypothesis. Moreover, in agreement with a meta-analysis on social desirability distortion [32], socio-demographics by and large did not moderate the relationship between social desirability and self-reported health risk behaviors. The only exception was education, which moderated the impact of self-deceptive enhancement on self-reported behavior frequency. This unanticipated effect warrants further investigation. However, given the high number of moderator tests conducted, this one effect might well be due to chance. Furthermore, there were no notable differences among the correlations of different social desirability measures with self-reported health risk behaviors. In pattern and size, these correlations were in line with previous research [37,46]. A possible explanation for the lack of a noteworthy association between social desirability and self-reported health risk behaviors is that respondents provide accurate self-reports of even undesirable behaviors, because the online setting increases their perceived privacy. An interviewer-administered questionnaire, by contrast, requires disclosure in front of an interviewer: The resulting shame might make underreporting undesirable behaviors more likely [49].

The studies at hand are potentially limited because current behavior and behavior frequency were measured by single items. Multiple-item measures might be more prone to social desirability distortion, because they increase the saliency of the undesirable behavior by way of repetition. Thus, our main outcome that people with tendencies of socially desirable self-presentation report the same degree of undesirable health risk behaviors than people with fewer tendencies of socially desirable self-presentation might not hold if multiple-item measures of health risk behaviors were used. Future research needs to shed light on this issue.

A strong point of the work at hand is the size and diversity of the samples. In contrast to previous research [23-25], we used three different samples from two demographically heterogeneous online panels from two different countries, providing the opportunity for generalization across samples. Outcomes across these three studies were largely congruent, which speaks in favor of the robustness of our findings. Thanks to the large sample sizes, the confidence intervals of the effects regarding social desirability were narrow (Additional files 1 and 2; Table 3), indicating an accurate estimation of effects [50]. Another benefit of the studies at hand is that they were longitudinal. Assessing participants' tendencies to present themselves in a socially desirable manner and obtaining their self-reports on socially undesirable health risk behaviors was spread apart in time. Therefore, our measurements are unlikely to be distorted by participants' unintentional and intentional attempts at portraying themselves as consistent, as might have happened had we obtained both sets of data in the same session. Finally, we used three different measures (Marlowe-Crowne Scale, BIDR, SDS-17) of social desirability, which pleads to the robustness of our findings across measures.

Although social desirability was not found to be consistently related to self-reported health risk behaviors in web-based research, this does not imply that self-report measures are equal to bio-medical measures in terms of validity. Previous research that compared self-report measures to bio-medical measures found mixed results. While predictions of urine drug screen had poor correspondence with self-report data [51,52], for example, there was a high consistency of self-report data with hair testing for drug use [53], a dipstick method assessing nicotine intake [54], and biological markers among alcohol-dependent patients [55]. This being only a general caution as this work was not about the comparative validity of self-reports versus bio-medical measures.

Furthermore, perhaps participants feared that their identity might be revealed by legal force, which possibly influences the validity of responses regarding illegal behavior (i.e., drug use). However, this fear would probably have led to more socially desirable responding, while the studies at hand revealed no meaningful associations between participants' self-reports and social desirability.

Last but not least, five final points need to be made. (1) Social desirability bias is not the only source of measurement error. Recall error, for example, may also lead to measurement error as may question format [56]. (2) There was mild selective drop-out in all studies. Those who dropped-out, for example, were younger than retainees. First, a certain level of drop-out is ubiquitous in longitudinal research, also on the web [57]. Second, the dropout in these studies seems to be innocuous, because socio-demographics did not moderate the impact of social desirability on self-reported health risk behaviors. (3) It is possible that some respondents might not have perceived alcohol use, drug use, and smoking as socially undesirable. Hence, they had no reason to tilt their self-reports into a favorable direction. However, this possibility alone can hardly account for the overall finding of a lack of a meaningful association between self-reported health risk behaviors and social desirability in as many as three samples. At any rate, future studies should examine the association between social desirability and self-reported health risk behaviors other than the ones looked at in the studies at hand. (4) These studies failed to find meaningful associations between social desirability and self-reported health risk behaviors. Because an absence of evidence of an association does not equal evidence of absence of an association, future research is not precluded from revealing such an association after all. However, the fact that the self-reports of different health risk behaviors were not considerably influenced by social desirability in as many as three studies that were longitudinal in nature and relied on large and heterogeneous samples gives us confidence in the robustness of our results. (5) This conclusion is backed up by the fact that in the three studies at hand that employed several measures of social desirability, a high number of statistical tests were conducted which entails a high likelihood of obtaining false positive results. Taking this inflation of Type I error into account, even the few small associations found between social desirability and self-reported health risk behaviors might well be due to chance.

## Conclusions

These studies do not throw doubt on the usefulness of the Internet as a medium to collect self-reports on health risk behaviors.

## Competing interests

The authors declare that they have no competing interests.

## Authors' contributions

Both authors substantially contributed to the conception and design of the study, and interpretation of data. RC drafted the manuscript and AG substantially contributed to revising it. Both authors approved the final version of the manuscript.

## Pre-publication history

The pre-publication history for this paper can be accessed here:

http://www.biomedcentral.com/1471-2458/10/720/prepub

## Supplementary Material

Additional file 1 **Results Study 1**. Effect of social desirability on self-reported health risk behaviors (Study 1; *N = *5,612). Click here for file

Additional file 2 **Results Study 3**. Effect of social desirability on self-reported health risk behaviors (Study 3; *N *= 846). Click here for file
