# Supplementary material for: Social desirability and self-reported health risk behaviors in web-based research: three longitudinal studies
Source: BMC Public Health. 2010 Nov 23;10:720. doi: 10.1186/1471-2458-10-720 (PMC2996374; doi:10.1186/1471-2458-10-720)
Supplement: Additional file 1 — Results Study 1. Effect of social desirability on self-reported health risk behaviors (Study 1; N = 5,612). [file 1471-2458-10-720-S1.DOC]

Additional file 1

Title: Results Study 1

Description: Effect of social desirability on self-reported health risk behaviors (Study 1; *N =* 5,612)

|  | Current behavior^1^ | | | | | | | Frequency^1^ | | | | | | |
| --- | --- | --- | --- | --- | --- | --- | --- | --- | --- | --- | --- | --- | --- | --- |
|  | Alc | Sed | SoD | XTC | Hal | HaD | Smo | Alc | Sed | SoD | XTC | Hal | HaD | Smo |
| Predictors | *d* | *d* | *d* | *d* | *d* | *d* | *d* | ß | ß | ß | ß | ß | ß | ß |
| Age | -.01* | .02* | -.03* | -.05* | -.04* | -.04* | .02* | .35* | -.16* | -.09 | .32 | -.11 | .02 | .17* |
| Sex | .37* | .34* | -.61* | -.40 | -.85* | -.64* | .12* | -.09* | .05 | .01 | -.29 | -.57 | -.30 | -.09* |
| Income | .00 | .00 | .00* | .00 | .00 | .00 | .00 | -.02 | .03 | .11 | .14 | -.20 | .17 | -.04 |
| Education^2^ | 51.43* | 11.31* | 1.92 | 1.65 | 1.88 | 2.57 | 57.64* | .08* | .09 | .07 | .07 | -.48 | -.13 | -.06* |
| SocDes^3^ | -.02 | .01 | .06 | .29 | .08 | .87* | .01 | -.01 | .24 | .02 | -.44 | .27 | -.06 | -.10 |
| Age × SocDes | .00 | .00 | .00 | .01 | .00 | .01 | .00 | -.01 | -.10 | -.14 | -.08 | .08 | -.24 | .03 |
| Sex × SocDes | -.03 | .02 | -.02 | .03 | -.03 | -.07 | .00 | .02 | -.10 | .12 | -.03 | -.40 | -.05 | -.01 |
| Income × SocDes | .00 | .00 | .00 | .00 | .00 | .00 | .00 | -.03 | .14 | -.07 | .06 | .16 | -.04 | -.01 |
| Education × SocDes^2^ | 7.85 | 4.35 | 1.07 | 2.22 | 1.55 | 6.28 | 1.82 | -.04 | -.21 | -.06 | .74 | -.02 | -.43 | -.13* |
| R^2^ | .08 | .06 | .15 | .14 | .26 | .17 | .09 | .14 | .08 | .06 | .37 | .64 | .30 | .05 |

^1^Alc = Alcohol use; Sed = Sedatives; SoD = Soft drugs; Hal = Hallucinogens; HaD = Hard drugs; Smo = Smoking; ^2^Wald statistic instead of Cohen’s *d;* ^3^Social desirability; * *p* < .05
