# Supplementary material for: Social desirability and self-reported health risk behaviors in web-based research: three longitudinal studies
Source: BMC Public Health. 2010 Nov 23;10:720. doi: 10.1186/1471-2458-10-720 (PMC2996374; doi:10.1186/1471-2458-10-720)
Supplement: Additional file 2 — Results Study 3. Effect of social desirability on self-reported health risk behaviors (Study 3; N = 846). [file 1471-2458-10-720-S2.DOC]

Additional file 2

Title: Results Study 3

Description: Effect of social desirability on self-reported health risk behaviors (Study 3; *N* = 846)

|  | BIDR-IM1^1^ | | | | SDS-17^1^ | | | | BIDR-IM2^1^ | | | | BIDR-SE^1^ | | | |
| --- | --- | --- | --- | --- | --- | --- | --- | --- | --- | --- | --- | --- | --- | --- | --- | --- |
|  | Current behavior | | Frequency | | Current behavior | | Frequency | | Current behavior | | Frequency | | Current behavior | | Frequency | |
|  | Alc | Smo | Alc | Smo | Alc | Smo | Alc | Smo | Alc | Smo | Alc | Smo | Alc | Smo | Alc | Smo |
| Predictors | *d* | *d* | ß | ß | *d* | *d* | ß | ß | *d* | *d* | ß | ß | *d* | *d* | ß | ß |
| Age | .00 | .00 | .23* | .13 | .00 | .00 | .26* | .10 | .00 | .00 | .25* | .10 | .00 | .00 | .20* | .11 |
| Sex | -.24* | .02 | -.17* | -.03 | -.18 | .06 | -.19* | -.03 | -.31* | .05 | -.17* | -.03 | -.27* | -.03 | -.20* | -.03 |
| Education^2^ | 20.40* | 34.31* | .05 | -.13* | 12.92* | 34.90* | .05 | -.11 | 9.69 | 26.99* | .06 | -.14 | 16.54* | 33.21* | .07 | -.15* |
| SocDes^3^ | .00 | -.55 | -.23 | .11 | .00 | .00 | .23 | .51 | .96 | .02 | -.18 | .03 | -.71 | -.08 | -.63* | -.67* |
| Age × SocDes | .00 | .00 | .03 | -.12 | .00 | .00 | -.05 | -.13 | .00 | .00 | -.03 | -.15 | .00 | .00 | -.03 | -.03 |
| Sex × SocDes | .07 | .13 | .00 | -.09 | .00 | .00 | -.11 | -.09 | .00 | .12 | -.01 | -.20 | -.03 | .11 | -.09 | -.03 |
| Education × SocDes^2^ | 2.73 | 5.72 | .11 | -.12 | 3.08 | 2.57 | -.36 | -.43 | 5.53 | 6.63 | -.04 | .13 | 3.86 | 1.80 | .68* | .69* |
| R^2^ | .08 | .09 | .11 | .05 | .06 | .10 | .14 | .06 | .07 | .11 | .14 | .06 | .07 | .10 | .13 | .07 |

^1^Alc = Alcohol use; Smo = Smoking; ^2^Wald statistic instead of Cohen’s *d;* ^3^Social desirability; * *p* < .05
